# Prep1 Deficiency Affects Olfactory Perception and Feeding Behavior by Impairing BDNF-TrkB Mediated Neurotrophic Signaling

**DOI:** 10.1007/s12035-018-0873-7

**Published:** 2018-01-18

**Authors:** Serena Ricci, Davide Viggiano, Ilaria Cimmino, Giuseppe Perruolo, Serena Cabaro, Antonietta Liotti, Francesca Fiory, Rosa Spinelli, Angelina Di Carlo, Francesco Beguinot, Pietro Formisano, Francesco Oriente

**Affiliations:** 1 Department of Translational Medical Science, “Federico II” University of Naples and URT “Genomics of Diabetes”, Institute of Experimental Endocrinology and Oncology of CNR, Via Pansini 5, 80131 Naples, Italy; 2 Department of Medico-Surgical Sciences and Biotechnologies, University of Rome “La Sapienza”, Latina, Italy; 3 Department of Medicine and Health Science, University of Molise, Campobasso, Italy

**Keywords:** Prep1, Olfactory bulb, BDNF, TrkB, Calbindin

## Abstract

Prep1 is a homeodomain transcription factor which has an important role in hindbrain development. Prep1 expression is also kept in adult mouse brain and in particular within the olfactory bulbs. Moreover, many Prep1 neurons co-localize with Calbindin-positive periglomerular interneurons in olfactory glomerular layer. However, Prep1 function in this brain region is still unknown. In this study, we show that *Prep1* hypomorphic heterozygous (*Prep1*^*i/+*^) mice express low levels of protein and feature a 30% reduction of olfactory bulb area, compared to WT mice. In addition, *Prep1*^*i/+*^ mice olfactory bulb histological analysis indicated a 20% lower cytochrome *C* oxidase activity within the glomerular layer, accompanied by a reduced number of periglomerular interneurons, compared to the WT littermates. Consistently, olfactory perception test highlighted that *Prep1* hypomorphic heterozygous mice display a scant ability to distinguish odors, which significantly impacts on feeding behavior, as *Prep1*^*i/+*^ mice revealed a reduced preference for high-fat food. Analysis of BDNF signaling, which represents the main molecular mediator of olfactory plasticity, showed that *Prep1*^*i/+*^ mouse olfactory bulbs feature a 30% reduction of TrkB receptor levels and a decreased activation of ERK1/2. Similarly, overexpression of Prep1 in mouse neuronal cells (N2A) caused an increase of TrkB expression levels, BDNF-induced ERK phosphorylation, and cell viability, compared to control cells. We conclude that Prep1 deficiency alters olfactory morpho-functional integrity and olfaction-mediated eating behavior by affecting BDNF-TrkB signaling. Prep1 could, therefore, play a crucial role in behavioral dysfunctions associated to impaired responsiveness to BDNF.

**Electronic supplementary material:**

The online version of this article (10.1007/s12035-018-0873-7) contains supplementary material, which is available to authorized users.

## Introduction

Chemical senses result from the evolutionary ability to recognize exogenous molecules and to develop appropriate responses. For the majority of species, including human, the chemical senses, and in particular smell, represent a vital function. Among all the existing animal species, there is no doubt that mouse is one with the most developed olfactory sense. In mouse, as well as in human, olfaction represents a critical sensory modality in regulation of exploratory and social approaching, but especially in regulation of eating behavior and peripheral metabolism [1–3]. Indeed, several clinical studies on obese patients observed a significant alteration of olfactory perception which can have an important impact on feeding habits [4, 5]. Nevertheless, notwithstanding the importance of the relation between olfaction and metabolic diseases, the genetic pattern involved in the regulation of olfactory-mediated feeding behavior remains largely unexplored.

Olfactory bulb (OB) is the first relay structure in olfactory processing, which receives direct input from olfactory sensory neurons (OSNs) in the olfactory epithelium and sends output to the olfactory cortex and other brain areas which originate neuroendocrine responses and innate behavior. The olfactory bulb circuitry encompasses two types of principal excitatory neurons, mitral and tufted cells, and two main inhibitory interneuron types, periglomerular (PGC) and granule cells. The cell bodies and dendrites of these neurons are organized into layers. The most superficial layer is composed of spherical structures called glomeruli in which the axons of OSNs make glutamatergic synapses with primary dendrites of mitral/tufted cells and PGC cells [6]. A unique characteristic of the olfactory sensory system, described in all vertebrates, is the continuous replacement of the olfactory neurons during the lifetime. Within the OBs, there is a substantial amount of newborn neurons which originate from progenitor cells in the subventricular zone (SVZ) and migrate along the rostral migratory stream (RMS) to become functional interneurons in the OB [7]. The combination of genes that regulates proliferation and cell fate determination of SVZ precursors remains to be identified, although neurogenesis steps have been described to be regulated in large part by neurotrophic factors, such as the insulin-like growth factor 1 (IGF-1) [8] and the brain-derived neurotrophic factor (BDNF). In particular, BDNF promotes migration, survival, and differentiation of newborn cells integrated in the OB by acting on tropomyosin receptor kinase B (TrkB) downstream receptors [9]. Previous studies demonstrated that newly generated neurons in olfactory bulb participate in fine odor discrimination, and that BDNF-haploinsufficient or TrkB-haploinsufficient mice do not spontaneously discriminate between odorants, suggesting that BDNF activity significantly impacts olfactory abilities [10]. However, these data remain, in part, correlative and the detailed mechanisms underlying these BDNF-related olfactory deficits are still unknown.

Prep1/Pknox1 is a member of transcription factors TALE family, which has recently been observed to have important effects on metabolic homeostasis [11–14]. However, Prep1 plays also a central function in the early and late stages of rodent brain development, by forming binary and ternary complexes with other homeodomain factors, such as Pbx1, Hoxb1, and Meis [15–19]. Stable expression and activity of these Prep1 co-factors, however, have been detected also in many regions of adult brain, especially in olfactory sensory areas [20–22]. In silico gene expression data, reported on Bio-GPS® (http://biogps.org/#goto=genereport&id=18771) and Allen Brain® (http://mouse.brain-map.org/gene/show/18535) web-based atlases, show that Prep1 is widely expressed in several regions of adult mouse brain but in particular within the olfactory bulb. However, unlike other TALE factors, no data about Prep1 function in olfactory brain regions and whether Prep1 misexpression may affect neurogenesis-dependent olfactory activity are to date available.

In the present work, we show that Prep1 represents a key factor in mouse olfactory bulb structural integrity which can strongly influence olfactory-related behaviors. These effects are related to an impairment of olfactory periglomerular interneuron replacement, which is paralleled by a reduced responsiveness of neuronal cells to BDNF-TrkB-mediated neurotrophic stimulation.

## Materials and Methods

### Materials

Media, sera, antibiotics for cell culture, lipofectamine reagent, Optimem, and Superscript III Reverse Transcriptase were from Invitrogen (Grand Island, NY). Prep1 plasmid cDNA (pRc/CMV-*Prep1*) has been designed in the laboratory and produced by Invitrogen (Grand Island, NY). The Prep1, BDNF, TrkB, ERK1/2, and 14-3-3θ antibodies were from Santa Cruz Biotechnology, Inc. (Santa Cruz, CA). The p-ERK1/2(Thr202/Tyr204) antibody was from Cell Signaling Technology, Inc. (Danvers, MA). Calbidin D-28k antibody was from Swant® (Marly, CH). S-100 antibody was from Ventana Medical System Inc. (Tucson, Arizona, USA). Protein electrophoresis and real-time PCR reagents were purchased from Bio-Rad (Hercules, CA), and Western blotting and ECL reagents from Amersham Biosciences (Arlington Heights, IL). Mayer Hemalum staining was from Bio-Optica S.p.A. (Milan, IT). Tissue-Tek OCT was from Sakura Finetek USA, Inc. (Torrance, CA). Recombinant human/mouse/rat BDNF protein was from Peprotech (Rocky Hill, NJ, USA). Cytochrome *C*, bovine catalase, DAB, sulforhodamine B, Eukitt, and all other chemicals were from Sigma-Aldrich (St. Louis, MO).

### Animals

C57BL/6J *Prep1* hypomorphic (*Prep1*^*i/+*^) mice were generated by gene-trapping by Lexikon Genetics, Inc. (The Woodlands, Texas). C57BL/6J *Prep1*^*i/+*^ mice and C57BL/6J wildtype (WT) littermates were housed two to three per cage at constant temperature and relative humidity, and were acclimated to a 12-h light/dark cycle and had ad libitum access to food and water. All animal procedures were approved by the Ethics Committee on Animal Use of University of Naples “Federico II” (permission number 363/2016-PR, prot. 39F3A.0) and comply with the standards of the European Union.

### Anatomical Analysis of Mouse Brain

Macromorphological analysis of brain structural alterations has been performed in 14 (7 *Prep1*^*i/+*^ and 7 WT) male 6-month-old mice. Mice have been euthanized and perfused with phosphate-buffered saline (PBS). The brains have been rapidly resected and fixed in 4% paraformaldehyde for 1 h at room temperature. Images were obtained by Chemidoc (Bio-Rad®), and morphological differences were analyzed by ImageJ® software, adding a scale bar of 3 mm.

### Hemalum and Cytochrome *C* Oxidase (COX) Staining

Olfactory bulbs (OBs) extracted from seven *Prep1*^*i/+*^ and seven WT male 6-month-old mice were fixed in 4% paraformaldehyde for 1 h at room temperature, cryoprotected in a 20% sucrose solution for 2 h, and embedded in OCT (optimal cutting temperature) compound prior to frozen sectioning on a microtome cryostat. OBs 20 μm coronal cryosections were incubated with Mayer Hemalum staining solution for 10 min at room temperature, rinsed with H_2_O for 5 min, dehydrated in ethanol decreasing scale, and soaked in xylene. Slices were mounted in Eukitt, and images were obtained with digital microscope camera (Leica®) (original magnification ×10). Images were analyzed by ImageJ® software, adding a scale bar of 50 μm. For COX activity evaluation, OBs 20 μm coronal cryosections were incubated at 37 °C for 2 h in a staining solution containing 24.4 mg of bovine cytochrome *C*, 125 mg of 3,3′-diaminobenzidine (DAB), 4.5 g of sucrose, 1 mL dimethyl sulfoxide (DMSO), and 50 μL of bovine catalase in 0.1 M pH 7.4 HEPES buffer. Sections were then incubated in 4% paraformaldehyde for 1 h at room temperature, rinsed with PBS, dehydrated in ethanol, and soaked in xylene. Slices were covered with a glass coverslip and images were obtained with a digital microscope camera (original magnification ×5). ImageJ® program was used for images analysis adding a scale bar of 100 μm. Images were quantitatively analyzed using ImageJ® software freely available at http://imagej.en.softonic.com. For COX activity quantification, the mean optical density (defined as – log (mean gray level/256)) was measured at the level of the glomerular and the external plexiform layer. For cell count measurement, images were converted to 8-bit, thresholded, segmented using a watershed filter, and the number of particles automatically counted using the “Analyze particles” plug-in. Periglomerular cell number is expressed as the number of cells per area (mm^2^), considering as ROI the glomerular layer.

### Immunofluorescence and Confocal Microscopy

Adult male *Prep1*^*i/+*^ and WT mice (*n* = 7 per group) have been deeply anesthetized and the brains fixed by trans-cardiac perfusion with 4% paraformaldehyde in PBS. Brains have been post-fixed for 2 h, then washed in PBS and cryopreserved in 20% sucrose in PBS overnight. After rapid freezing in dry ice, 80-μm-thick free-floating sections have been cut in the sagittal plane with a cryostat and collected in PBS in a multi-well plate. Sections were then incubated overnight at 4 °C with one of the following primary antibodies: (1) rabbit anti-Calbindin (1:1000) + mouse anti-Prep1 (1:100), (2) rabbit anti-TrkB (1:100), and (3) mouse anti-S-100 (1:100). Primary antibodies were diluted in PBS + 10% bovine serum. Sections were then washed in PBS three times and incubated with donkey anti-rabbit-CY3 (1:100) + donkey-anti-mouse-CY2 (1:100) secondary antibodies (Jackson ImmunoResearch Europe Ltd., Suffolk, UK) diluted in PBS + 10% bovine serum for 1 h at room temperature; afterwards, sections were washed in PBS three times and finally mounted in PBS/glycerol (1:1). Images were acquired with a Zeiss LSM 510 confocal laser scanning microscope, using 488 and 543 nm excitation wavelengths and, sequentially, a BP 505–550 emission filter for CY2 and LP560 emission filter for CY3. Images were acquired with different magnifications to show different brain structures (×10 objective) or cellular components (×63 objective), with a resolution of 1024 × 1024 pixels and zoom factors up to ×4.

### Behavioral Tests

Behavioral monitoring was performed on male *Prep1*^*i/+*^ (*n* = 9) and WT littermates (*n* = 9) at the age of 6 months, during the light phase between 10:00 a.m. and 5:00 p.m. A modified SHIRPA protocol has been used as a first screening to observe gross abnormalities in posture and sensorial response. The presence of foot clasping (abduction of hind limbs) has been evaluated suspending mice by their tails. Subsequently, mice have been tested for their negative geotaxis and climbing in order to underline alterations in motor abilities. A minimum of 1 day separated the testing sessions.

### Open Field

Mice were placed in the middle of a clear Plexiglas (50 × 50 × 25 cm^3^) chamber and allowed to explore the novel environment for 5 min. The arena has been subdivided in nine square regions and the number of entries in adjacent square regions was counted offline as the index of total locomotor activity. The number of entries in the central region (30 × 30 cm^2^) was also separately measured. The number of entries in adjacent square regions minus the number of entries in the central region ratio was considered as an index of anxious-like behavior.

### Olfactory Preference Test

To assess olfaction, olfactory perception test was performed on 12-h fasting *Prep1*^*i/+*^ and WT mice. One hour prior to test, mice have been habituated by placing their cage in the testing room with no water bottle. Animals were placed in the clean assigned cage and let them explore for 5 min until the environment of the experimental cage was familiar to the home cage (habituation). The odorant stimuli were tap water (neutral odor), cinnamon in H_2_O (1% *w*/*v*), and peanut butter in peanut oil (10% *w*/*v*). These two odor scents were selected as favorite among nine different odor scents (i.e., orange, lemon, vanilla, strawberry, banana, cinnamon, lavender, peanut, and mint) based on preliminary novel odor recognition trials with other C57BL/6J mice (data not shown). After habituation, immediately 20 μL of selected scents (cinnamon or peanut butter) and 20 μL of the neutral scent (tap water) were spotted on blotting paper (5 × 5cm) and placed onto the opposite walls of the cage. Mice were allowed to explore the odors for a total of 3 min. An experimenter recorded the cumulative time that the mice spent sniffing the different scents. Measurement of olfactory perception was performed by comparing the times (s) spent with the odors subtracted to the time spent with water between the genotypes.

### Food Preference Test

For the food preference assessment, during each of four pre-test days, *Prep1*^*i/+*^ and WT mice were given continuous access in the home cage to both high-fat food and standard food pellets (60% kcal% fat and 10% kcal% fat, respectively; Research Diets Inc., Brunswick, NJ). On the fifth day, all animals were food deprived for 12 h and then given a choice between the same two foods in a 12-h preference test. Consumption of each foods was recorded for all mice. Preference index was calculated according to following ratio: grams of standard or high fat food eaten/total grams of food (standard + high fat) eaten.

### Cell Culture Procedures and Transfection

Mouse neuroblastoma cells (N2A) were cultured at 37 °C in Dulbecco’s modified Eagle’s medium (DMEM) supplemented with 10% fetal bovine serum (FBS), 2% l-glutamine, 10,000 U/mL penicillin, and 10,000 g/mL streptomycin. Transient transfection of Prep1 plasmid cDNA (pRc/CMV-*Prep1*) was performed by using Lipofectamine 2000 reagent according to the manufacturer’s instruction. For these studies, 60–80% confluent cells were washed twice with Optimem and incubated for 8 h with 2–5 g of plasmid construct and 6–15 μL of lipofectamine reagent. The medium was then replaced with DMEM with 10% FBS and cells further incubated for 15 h before being assayed. For BDNF stimulation, N2A cells were starved overnight and stimulated with BDNF (50 ng/mL) for 10 min or 24 h.

### Western Blotting

Tissue samples were homogenized in a Polytron (Brinkman Instruments, NY) in 20 mL T-PER reagent/g of tissue according to the manufacturer (Pierce, IL). After centrifugation at 10,000 rpm for 5 min, the supernatant was collected. Cells were solubilized in lysis buffer (50 mmol/L HEPES, pH 7.5, 150 mmol/L NaCl, 10 mmol/L EDTA, 10 mmol/L Na_4_P_2_O_7_, 2 mmol/L Na_3_VO_4_, 100 mmol/L NaF, 10% glycerol, 1% Triton X-100, 1 mmol/L PMSF, 10 mg/mL aprotinin) for 1 h at 4 °C and lysates were centrifuged at 14,000 rpm for 20 min. Total homogenates were separated by SDS-PAGE and transferred on 0.45-m Immobilon-P membranes. Upon incubation with primary and secondary antibodies, immunoreactive bands were revealed by an enhanced luminol-based detection (electrochemiluminescence, ECL), according to the manufacturer’s instructions (Amersham®; GE Healthcare, Uppsala, Sweden) and the autoradiographs were subjected to densitometric analysis.

### Real-Time (RT-PCR) Analysis

Total RNA was isolated from brain tissue and N2A cells by using the QIAGEN RNeasy kit (QIAGEN Sciences, Germany) according to the manufacturer’s instructions. One microgram of tissue or cell RNA was reverse-transcribed using Superscript III Reverse Transcriptase. PCR reactions were analyzed using SYBR Greenmix (Bio-Rad®, Hercules, CA). Reactions were performed using Platinum SYBR Green qPCR Super-UDG using an iCycler IQ multicolor Real-Time PCR Detection System (Bio-Rad®, Hercules, CA). All reactions were performed in triplicate and *β-actin* was used as an internal standard. Primer sequences used were as follows: human/mouse Prep1 F: 5′**-**GGAGTGCCAACCATGTTAAGAAGAAGTCCC-3′, R: 5′-GACACCGTGTGCTTCTCGCTCAAG-3′; mouse TrkB F: 5′-TCACTTCGCCAGCAGTAGC-3′, R: 5′-CTCAGGGCTGGGGAGCAAC-3′; mouse β-actin F: 5′-CGCCCTAGGCACCAGGGTGTG-3′, R: 5′-TCGGTGAGCAGCACAGGGTG-3′.

### Cell Viability Assay

N2A cells were seeded in 12-well culture plates at a concentration of 5 × 10^4^ cells/mL in a complete medium and transiently transfected with Prep1 plasmid cDNA. After an overnight starvation from serum, cells were stimulated with BDNF (50 ng/mL) for 24 h and cell viability was assessed by sulforhodamine B assay. Briefly, the cells were fixed with 50% trichloroacetic acid for at least 2 h at 4 °C. Then, cells were washed five times with distilled and deionized water. After air-drying, cells were stained for 30 min with 600 μL 0.4% sulforhodamine B dissolved in 1% acetic acid. Unbound dye was removed by five washes with 1% acetic acid. After air-drying, 10 mM Tris solution (pH 7.5) was added to dissolve the protein-bound dye. Cell density was assessed by optical density determination at 510 nm using Infinite® 200 PRO plate-reader (Tecan Trading AG, Switzerland). Three replicate wells were used for each data point. Each experiment was performed three times.

### Statistical Procedures

Data were analyzed with the GraphPad Prism 5.04 software (GraphPad Inc., San Diego, CA, USA); comparisons between *Prep1*^*i/+*^ and WT mice, and between N2A and N2A^Prep1^cells were performed via two-tailed Student’s *t* test for unpaired data. *p* values equal or less than 0.05 were considered statistically significant.

## Results

### Prep1 Expression in Mouse Central Nervous System

**Fig. 1:**
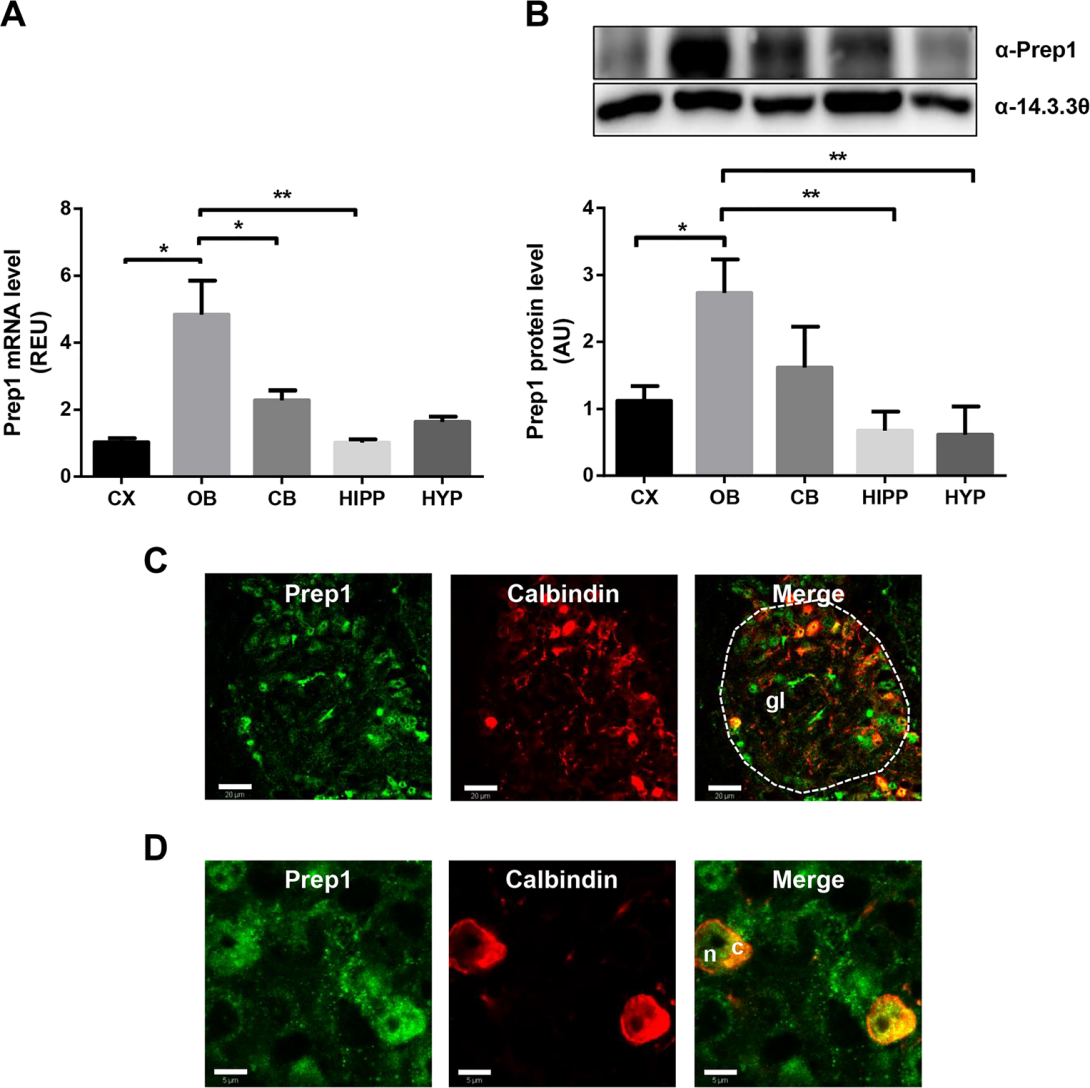
Prep1 levels in adult C57BL/6J mouse brain regions. **a** Prep1 mRNA levels in the different brain regions of 6-month-old C57BL/6J mice. Bar represents the mean ± SEM of three independent experiments, in each of which reactions were performed in triplicate using the pooled total RNAs obtained from five mice. **b** Prep1 protein levels in brain regions from C57BL/6J mice. The autoradiographs are representative of four independent experiments. Asterisks denote statistically significant differences (*p < 0.05, **p < 0.01). **c**, **d** Immunofluorescence staining for Prep1 (green) and Calbindin (red) in the olfactory bulb at ×20 (**c**; scale bar, 20 μm) and ×63 (**d**; scale bar, 5 μm) magnification. The contour of a glomerulus (Gl) is indicated with a dashed line in (**c**). Periglomerular cells in (**d**) are shown with their Prep-1-ir nuclei (n) and Prep1/Calbindin-ir cytoplasm (c). CX, cortex; CB, cerebellum; c, cytoplasm; Gl, glomerulus; HIPP, hippocampus; HYP, hypothalamus; n, nucleus; OB, olfactory bulb

In order to confirm in silico Prep1 expression data, reported on Allen Brain Atlas® and BioGPS® atlases, RT-PCR and western blot analysis of 6-month-old C57BL/6J mouse brain was performed. As shown in Fig. 1, olfactory bulb (OB) represents the brain region with the highest level of Prep1 mRNA (Fig. 1a) and protein (Fig. 1b), compared to other regions including cerebral cortex (CX), cerebellum (CB), hippocampus (HIPP), and hypothalamus (HYP). Double immunofluorescence staining for Prep1 and a marker of interneurons, Calbindin, shows that Prep1 is expressed at the level of the nucleus and partially in the cytoplasm of Calbindin-immunoreactive (Calbindin-ir) neurons (Fig. 1c, d). Compared to other brain regions, the staining intensity is highest in the olfactory bulb (Supplemental Fig. 1), with major concentration in Calbindin-ir periglomerular cells (Fig. 1c, d). Lower expression levels were found in Calbindin-ir neurons of superficial layers of the cortex (layers 2, 3), in the Purkinje cells of the cerebellum, in Calbindin-ir interneurons of the ventral part of the hindbrain, and in interneurons of the olfactory tubercle (Supplemental Fig. 1). On the other hand, immunostaining for Prep1 was found undetectable in the caudate putamen (striatum), thalamus, hypothalamus, hippocampus, and mesencephalic colliculi (Supplemental Fig. 1). Indeed, the distribution of Prep1 was more limited than Calbindin, as Calbindin-ir neurons in many brain regions do not express noticeable levels of Prep1 (e.g., in the striatum, the hippocampus, the thalamus, and the hypothalamus) (Supplemental Fig. 1). While in other brain regions the co-localization of Prep1 and Calbindin is high, in the olfactory bulb some cells do not co-express Prep1 and Calbindin. A quantification on 50 Calbindin-ir cells and 50 Prep1-immunoreactive (Prep1-ir) cells in the olfactory bulb showed that in WT animals 45% of the cells expressed both markers (Calbindin+/Prep1+), 46% expressed only Prep1 (Calbindin−/Prep1+), and 9% expressed Calbindin with very low levels of Prep1 (Calbindin+/Prep1−). Separate counts in *Prep1*^*i/+*^ mice showed similar percentage of co-localization (Calbindin+/Prep1+, 45%; Calbindin-/Prep1+, 44%; Calbindin+/Prep1−, 10%), which was not statistically different from WT animals (chi-square test, *p* = 0.9). We have not identified as yet the type of cells expressing Prep1 but not Calbindin, although their anatomical localization corresponds to some other subtype of periglomerular cells.

### Morphological Analysis of *Prep1*^*i/+*^ Mouse Brain

**Fig. 2:**
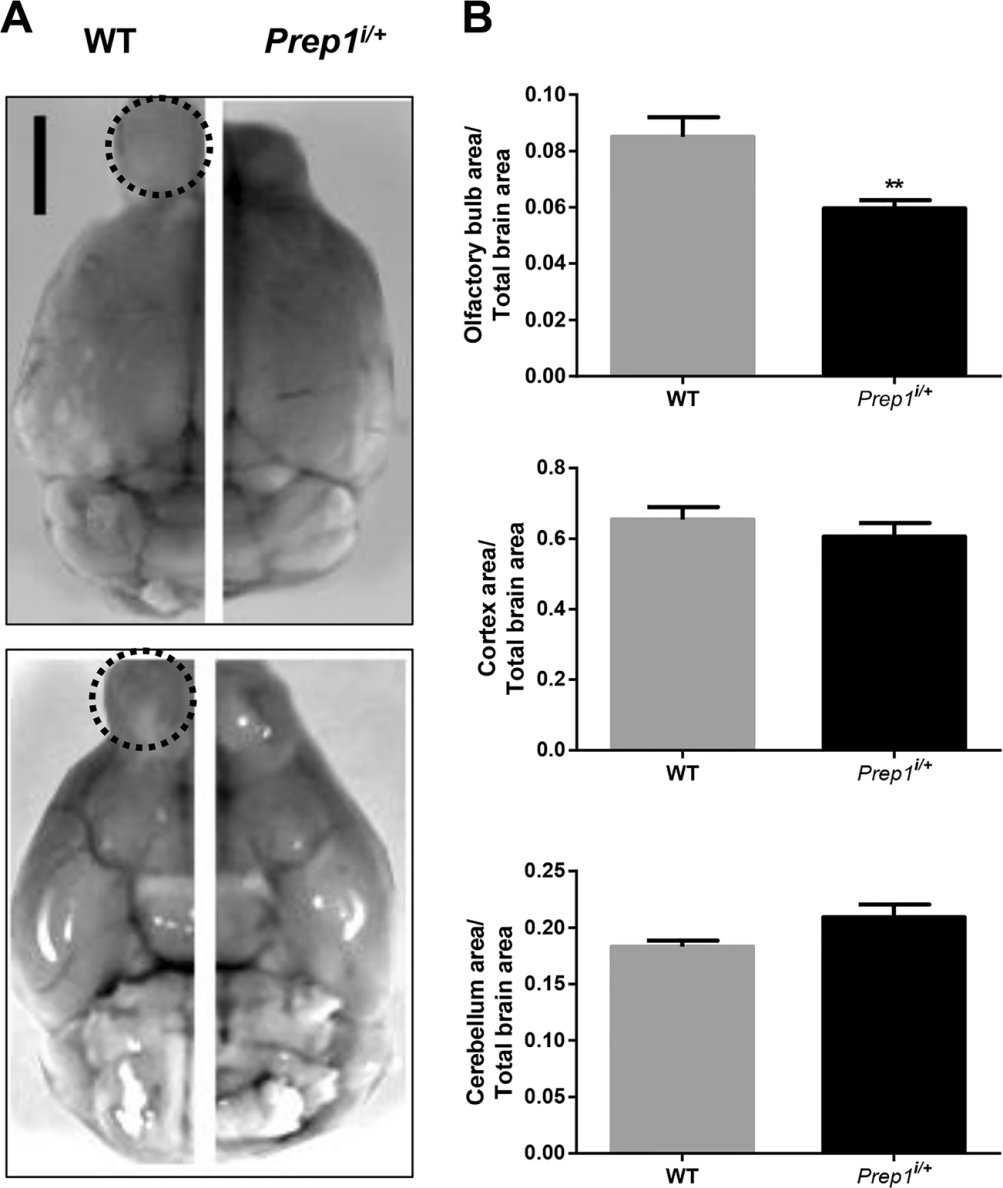
Anatomical analysis of *Prep1*^*i/+*^ mice brain. **a** Macroscopic appearance of brains from WT and *Prep1*^*i/+*^ mice (scale bar, 3 mm). The olfactory bulb area is indicated by a dashed line. **b** Projection areas of the cerebral hemispheres, cerebellum, and the olfactory bulb were measured. Results are expressed as ratio of the brain region over the total brain area. Bar represents the mean ± SEM of seven mice per genotype. Asterisks denote statistically significant differences (**p < 0.01)

**Fig. 3:**
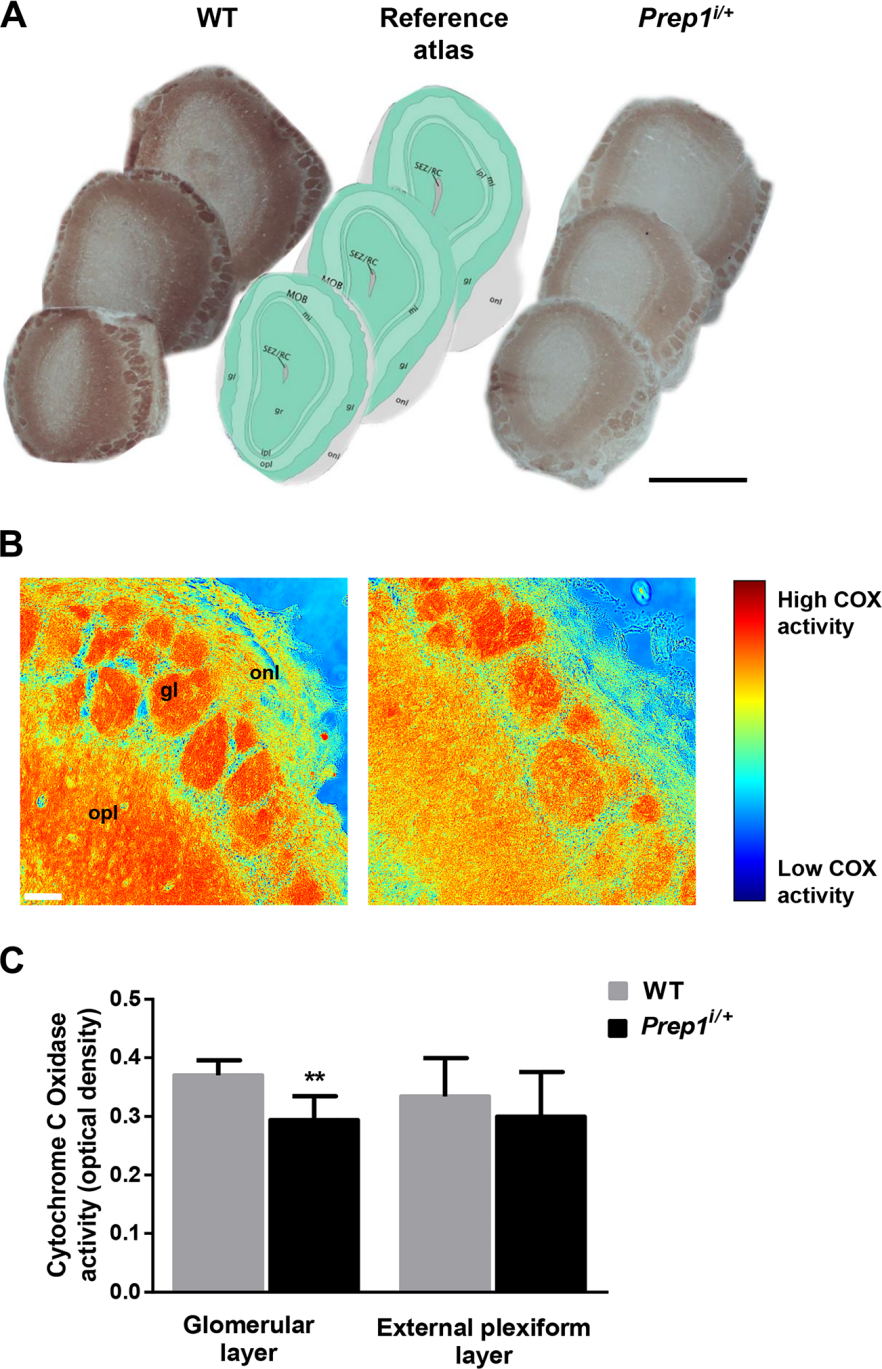
COX staining of *Prep1*^*i/+*^ mice OB sections. **a** Representative images of COX staining of olfactory bulb from *Prep1*^*i/+*^ and WT mice are shown (original magnification ×5; scale bar, 1 mm). Reference drawings have been adapted from Allen Brain Reference Atlas®. **b** Higher magnification (original magnification ×20; scale bar, 50 μm) of COX-stained OB sections represented with pseudo-colors to better show differences in staining intensity. gl, glomerular layer; epl, external plexiform layer. **c** Quantification of COX activity has been performed by ImageJ®. Bars represent mean ± SEM of COX optical density in different olfactory bulb layers. Asterisks denote statistically significant differences (**p < 0.01). gl, glomerular layer; gr, granular layer; opl, outer plexiform layer; ml, mitral layer; ipl, inner plexiform layer; onl, olfactory nerve layer; MOB, main olfactory bulb; SEZ/RC, sub-ependymal zone

To understand the role of Prep1 in OB, we performed a macromorphological evaluation of the total WT and *Prep1*^*i*/+^ mice brain structure. As shown in Fig. 2, anatomical analysis indicated that *Prep1*^*i/+*^ mice display a significant 30% reduction of OB area compared to WT mice. At variance, no significant differences were detected between the other structures (Fig. 2). Subsequently, OB cryosections obtained from WT and *Prep1*^*i/+*^ mice littermates were analyzed by cytochrome *C* oxidase (COX) staining in order to quantify neuronal metabolism. In particular, as COX activity has been described to be mainly present within the glomerular layer, where periglomerular cells axons and olfactory fibers converge, and in the external plexiform layer (EPL) where mitral and tufted cell axons converge [23], we quantified COX activity in these OB areas. As shown in Fig. 3, *Prep1*^*i/+*^ mice displayed a 20% reduction of COX activity within the glomerular layer compared to WT mice, while a not statistically significant reduction of COX activity was observed within the external plexiform layer (Fig. 3a–c).

**Fig. 4:**
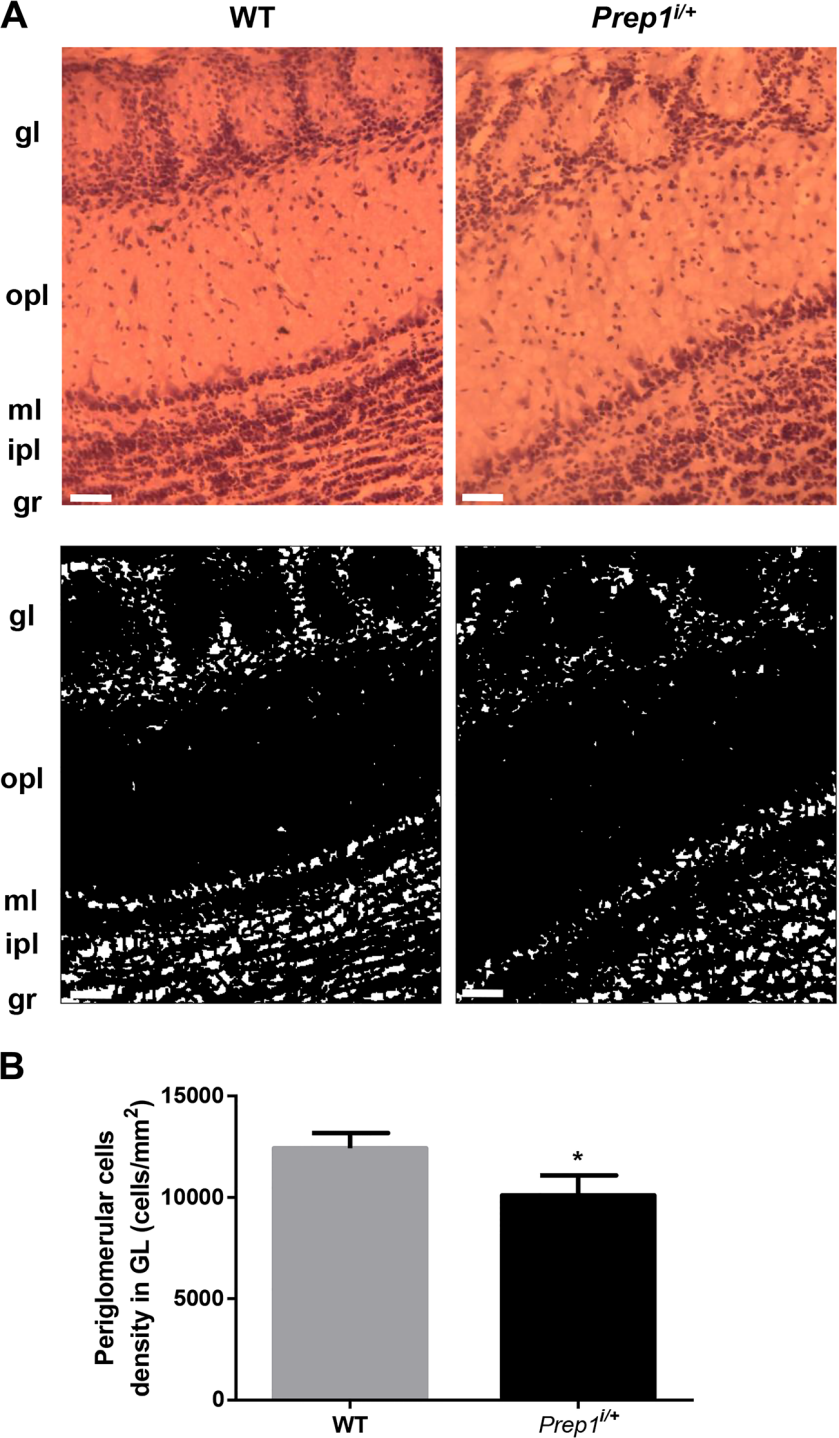
Hemalum staining of *Prep1*^*i/+*^ mice OB sections. **a** Representative images of olfactory bulb coronal cryosections from WT and *Prep1*^*i/+*^ mice stained with Hemalum staining, a solution of hematoxylin and alum able to stain cell nuclei (scale bar, 50 μm). A threshold was applied to the images in order to identify and count the nuclei (lower panel). **b** Quantification of neuronal density in seven *Prep1*^*i/+*^ and seven control animals. Asterisks denote statistically significant differences (*p < 0.05). gl, glomerular layer; gr, granular layer; opl, outer plexiform layer; ml, mitral layer; ipl, inner plexiform layer; onl, olfactory nerve layer

In parallel, we have also analyzed OB cryosections by Mayer Hemalum, a histochemical staining solution formed from aluminum ions and hematein (an oxidation product of hematoxylin) which is able to color nuclei of cells. *Prep1* hypomorphic heterozygous mice displayed a normal morphological structure of olfactory bulb with no significant difference in the width of the layers. However, histochemical staining revealed significant differences in the number of olfactory cells between two genotypes. Count density measurement showed significant 30% reduction of periglomerular cells in *Prep1*^*i/+*^ OB than that of WT mice. No significant differences in other OB layers were observed (Fig. 4).

Additional immunofluorescence staining for S-100-ir astrocytes in the olfactory bulb has been performed to verify the presence of astrogliosis, an indirect effect of neurodegeneration. As shown in Supplementary Fig. 3, no difference in S-100 staining was noted between WT and *Prep1*^*i/+*^ mice.

### Behavioral Monitoring of *Prep1* Hypomorphic Mice

In order to verify whether the reduced number and metabolism of olfactory periglomerular neurons might impair the olfactory function, we performed a behavioral monitoring of WT and *Prep1*^*i/+*^ littermates. To evaluate the susceptibility to depression-like state, a tail suspension test was performed. *Prep1*^*i/+*^ mice displayed a tail elevation comparable to WT littermates and showed a regular posture with extended hind limbs. Immobility time during suspension was similar among groups. Moreover, no differences in time to climb and negative geotaxis have been detected (data not shown). To examine locomotor activity and behavioral anxiety, an open field test was administered. A significant difference in active exploratory behavior was noted between the two genotypes. Indeed, the number of entries in the adjacent square regions was significantly lower in *Prep1*^*i/+*^ mice compared to WT littermates. *Prep1*^*i/+*^ mice also featured a slight reduction in center square region entries, albeit not reaching a statistical significance. No variance in center-to-total locomotor activity ratio was observed, indicating absence of differences in anxious-like behaviors between the groups (Supplemental Fig. 2).

**Fig. 5:**
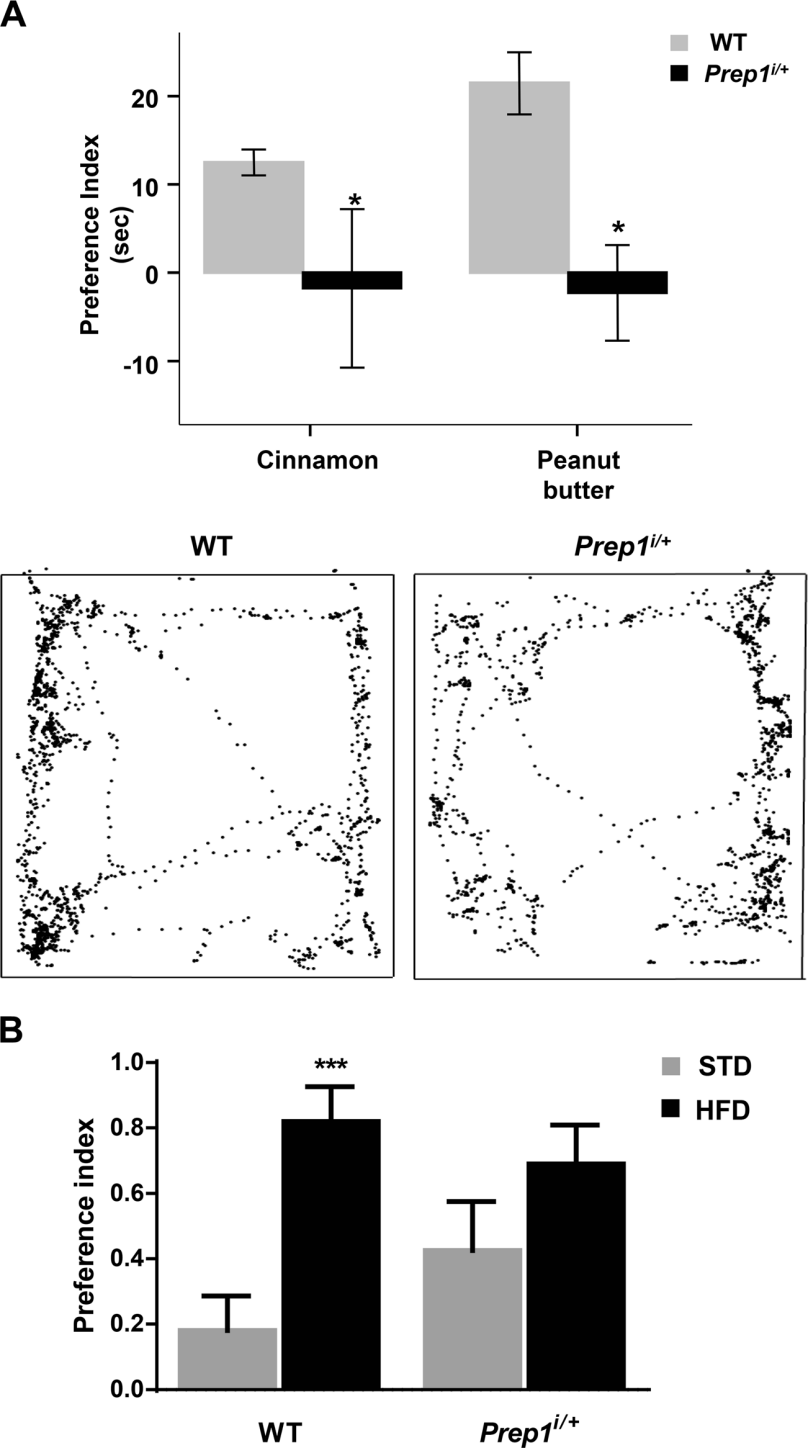
Olfactory and food preference in *Prep1*^*i/+*^ mice. **a** WT and *Prep1*^*i/+*^ mice (*n* = 9 per group) have been exposed to a 3-min preference test between an odorous (peanut butter or cinnamon) and a neutral scent (water), as described in the “Materials and Methods” section. Representative traces of animals exploring the arena, in which an odorous scent is placed in down-left corner and a neutral scent is placed in up-right corner, are shown. **b** WT and *Prep1*^*i/+*^ have been exposed to a 12-h preference test between high-fat and standard foods, as described in the “Material and Methods” section. Asterisks denote statistically significant differences (*p < 0.05, ***p < 0.001)

Subsequently, we analyzed olfactory detection abilities by performing olfactory perception test in order to measure the preference index to odorous scents (e.g., peanut butter and cinnamon). WT and *Prep1*^*i/+*^ mice were tested and the exploratory time to discover the odor items was quantified subtracting the time spent on odor to time spent on water (preference index). As shown in Fig. 5, the WT group displayed a higher preference to peanut butter and cinnamon than to water. On the contrary, *Prep1*^*i/+*^ mice showed a significantly very low attraction to cinnamon and to peanut butter, spending more time with water (Fig. 5a). Since olfaction has a prominent role in regulating appetite and eating choice [1–3], we evaluated whether the impaired olfactory discrimination ability observed in *Prep1*^*i/+*^ animals might influence feeding behavior. For determining the impact of altered olfaction in feeding choice, animals underwent a food preference test, able to measure the preference for high-fat food. WT mice displayed a strong and significant preference for high-fat food than for standard chow. Conversely, *Prep1*^*i/+*^ mice did not feature a statistically significant preference for high-fat food than standard one (Fig. 5b).

### Evaluation of BDNF-TrkB Signaling Pathway in *Prep1*^*i/+*^ Mice OB

**Fig. 6:**
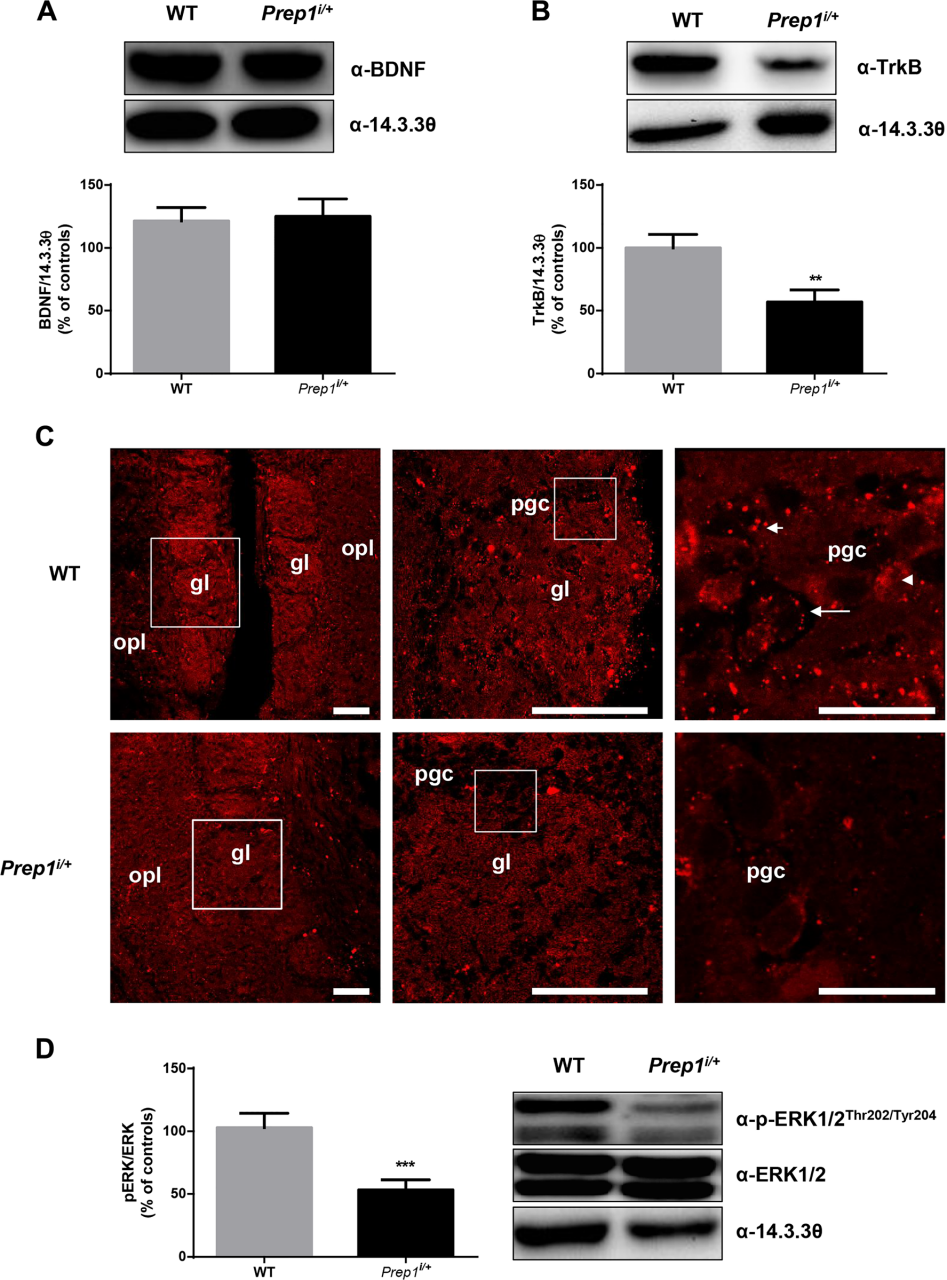
BDNF-TrkB signaling in *Prep1*^*i/+*^ mice OB. **a** BDNF protein levels in OBs from WT and *Prep1*^*i/+*^ mice. **b** TrkB protein levels from OBs from WT and *Prep1*^*i/+*^ mice. **c** Immunofluorescence for TrkB in WT and *Prep1*^*i/+*^ mice olfactory bulb has been analyzed by confocal microscopy as reported in the “Material and Methods” section; three different magnifications are shown to better appreciate the differences (scale bars, 50 μm for left and middle panels; 10 μm for right, high-magnification panels; squares indicate the position of magnified images). TrkB was localized onto the cell membrane (arrows), the cytoplasm (arrowheads), and in the neuropile (short arrows) in a punctate pattern. **d** p-ERK1/2 and ERK1/2 levels in OBs from WT and *Prep1*^*i/+*^ mice. 14-3-3θ antibody was used for normalization. The autoradiographs shown on the top of the graphic are representative of four independent experiments. Asterisks denote statistically significant differences (**p < 0.01, ***p < 0.001). gl, glomerular layer; opl, outer plexiform layer; pgc, periglomerular cells

Several evidences suggest a key role of BDNF signaling in regulating differentiation of periglomerular cells from neuroblasts within the subventricular zone (SVZ) (neurogenesis) and in affecting olfactory tuning and plasticity [24]. Thus, in order to verify the hypothesis that Prep1 might affect olfactory bulb neurons’ responsiveness to BDNF neurotrophic signals, we measured BDNF protein levels in OBs from WT and *Prep1*^*i/+*^ mice. As shown in Fig. 6, no significant difference was detected between the two genotypes (Fig. 6a). However, the expression levels of main target receptor for BDNF (TrkB) revealed that *Prep1*^*i/+*^ mice display a significant reduction of TrkB protein levels compared to WT mice (Fig. 6b). Immunostaining on OB sections from WT and *Prep1*^*i/+*^ mice further indicated that, within the glomerular layer, TrkB immunoreactivity was clearly lower in *Prep1*^*i/+*^ mice than WT littermates (Fig. 6c). We have quantified the fluorescence intensity by measuring the gray level (fluorescence intensity) of immunoreactive structures and the area covered by immunoreactive structures divided by the total area (proportional area). The results showed a 22% decrease in immunofluorescence intensity in *Prep1*^*i/+*^ mice (fluorescence intensity units (8-bit gray level), WT = 39 ± 2, *Prep1*^*i/+*^ = 30 ± 3; *p* = 0.05, Student’s *t* test), without modification in the proportional area (area covered by immunoreactive puncta, WT = 1.6 ± 0.3% of total area; *Prep1*^*i/+*^ = 1.6 ± 0.3%; *p* = 0.84). Therefore, the immunostaining intensity per cell was decreased, whereas the area covered by TrkB puncta (a surrogate for the number of stained cells) was not modified. Consistently, evaluation of phosphorylation levels of the main kinase described to be involved in BDNF/TrkB neurotrophic signaling pathway in OB [25] revealed that *Prep1*^i/+^ mice feature 50% reduction of p-ERK1/2 compared to control animals, with no difference in ERK1/2 total protein amount (Fig. 6d).

### BDNF-TrkB Neurotrophic Signaling in Neuronal Cells Overexpressing Prep1

**Fig. 7:**
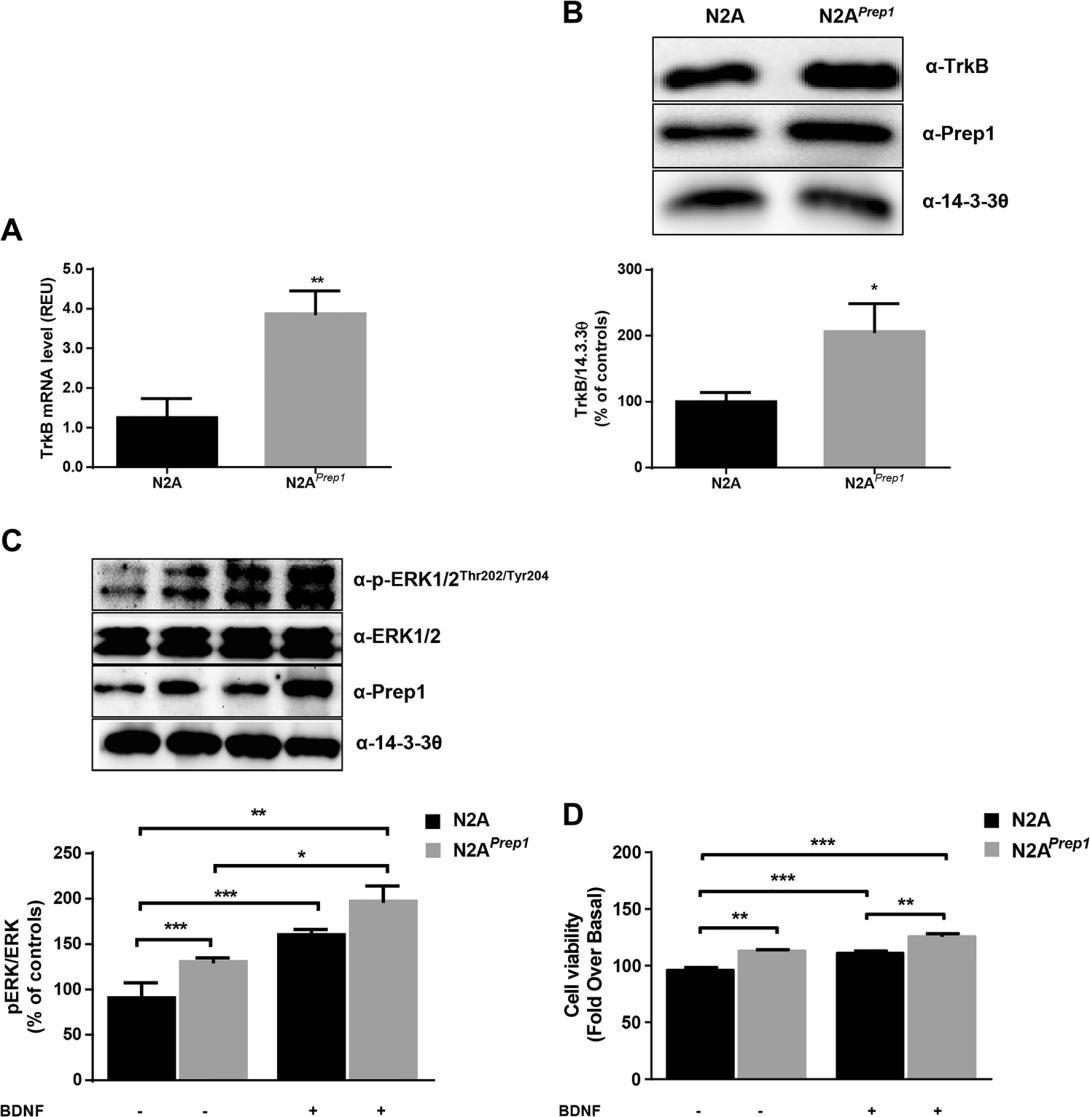
BDNF-TrkB signaling in N2A cells overexpressing Prep1. N2A cells were transiently transfected with Prep1 cDNA as described in the “Materials and Methods” section. **a** TrkB (*ntrk2*) mRNA in control and N2A^*Prep1*^ cells. Bar represents the mean ± SEM of three independent experiments. **b** TrkB protein levels in control and N2A^*Prep1*^ cells. 14-3-3θ antibody was used for normalization. The autoradiographs shown on the top of the graphic are representative of three independent experiments. **c** N2A^*Prep1*^ were stimulated with recombinant BDNF (50 ng/mL) for 10 min and protein samples were analyzed by Western blot with Prep1, p-ERK1/2, and ERK1/2 antibodies. 14-3-3θ antibody was used for normalization. The autoradiographs shown on the top of the graphic are representative of four independent experiments. **d** N2A^*Prep1*^ were stimulated with recombinant BDNF (50 ng/mL) for 24 h and cell viability was measured by sulforhodamine B assay as described in the “Materials and methods” section. Bars represent the mean ± SEM of three independent experiments, in each of which reactions were performed in quintuple. Asterisks denote statistically significant differences (*p < 0.05, **p < 0.01, ***p < 0.001)

To further investigate the effects of Prep1 on BDNF-mediated neurotrophic signaling, we transiently transfected a mouse neuronal cell line (N2A) with a *Prep1* cDNA (pRc/CMV-*Prep1*). Analysis of TrkB gene and protein levels showed a fourfold increase of TrkB transcript (Fig. 7a) and twofold increase of TrkB protein amount (Fig. 7b) in N2A overexpressing Prep1 (N2A^*Prep1*^) compared to control cells. In addition, ERK1/2 phosphorylation was observed significantly higher in N2A^*Prep1*^ cells both in basal conditions and after direct stimulation with recombinant BDNF protein (Fig. 7c). Consistently, cell viability was found also increased in a similarly sized manner (Fig. 7d).

## Discussion

Alteration in approaching to food is considered a fundamental aspect in metabolic unbalance and, in most cases, the psychological input for crossing the borderline which divides over-eating from binge-eating. An increasing literature defined a pivotal role of chemical senses, like smell and taste, in food appreciation and perception [2]. In particular, olfactory-mediated regulation of appetite for high-fat foods has been extensively studied in animal models and in human [26–29], even though no great progresses have been made in defining how much genetic background might impact on individual eating-related chemosensory landscape [30]. Prep1/Pknox1 transcription factor has been widely investigated in our previous studies as an important regulator of metabolic homeostasis, as Prep1-deficient mouse model displays better insulin sensitivity and a reduced risk of developing diabetes and diabetes-related comorbidities [11–14]. Although the expression of *Prep1* gene in adult mouse is mostly related to brain (MGI-Mouse Genome Informatics®, http://www.informatics.jax.org/expression.shtml), no previous evidences have clarified its tissue-specific function. According to information reported in web atlases (e.g., Allen Brain Atlas®, BioGPS®), as well as our protein and gene expression analysis, Prep1 expression in C57BL/6J mice is particularly focused in olfactory bulbs compared to other brain regions. Indeed, as shown by immunofluorescence, Prep1 is particularly enriched in periglomerular cells of the olfactory bulb. Prep1 is also expressed in other brain regions and generally shows a co-localization with Calbindin, a marker of interneurons. Specifically, Prep1 is expressed in slow-spiking interneurons of the cortex (marked by Calbindin), in Purkinje cells of the cerebellum, and in the olfactory tubercle and ventral region of the hindbrain, whereas other brain regions do not express Prep1 protein at detectable levels. We also observed that at cellular level, Prep1 is expressed mostly at nuclear level, in agreement with its role as transcription factor.

Previous studies demonstrated that many Prep1 co-factors, belonging to TALE family, are highly abundant in mouse olfactory bulbs and play key roles in olfactory interneurons’ regeneration from SVZ neuroblasts [21, 22]. Therefore, the marked expression of Prep1 in olfactory bulb might suggest a key biological function of this transcription factor in olfactory sensory circuit or olfactory renewal processes, which are inevitably related. Consistent with this hypothesis, anatomical comparison of WT and *Prep1*-deficient mice brains highlighted an impaired formation of olfactory bulb in *Prep1*^*i/+*^mice. Histological examination underlined also a significant difference in the cytoarchitecture of the olfactory bulb between *Prep1* hypomorphic heterozygous and WT mice, as *Prep1*^*i/+*^ mice showed a reduction in the number of adult-generated periglomerular cells (PGCs) which is accompanied by a significantly reduced cytochrome *C* oxidase activity in the glomerular layer. Within the olfactory bulb, the glomerular activity pattern is represented by the intricate interplay between output and input neurons, which defines a balanced circuit that allows odorant identity and recognition [31, 32]. Thus, it is plausible to hypothesize that the imbalance in olfactory bulb cells and, in particular, the substantial reduction of PGCs number and oxidative metabolism in *Prep1*^i/+^ mice might induce behavioral changes. Interestingly, olfactory perception test revealed that *Prep1*^i/+^ mice react differently from WT mice in their persistence in investigating attractive odors and in their ability to spontaneously discriminate between smells. Reduced response to odorous stimuli might be the result of disturbed olfaction probably due to changes in synaptic connectivity that diminish odor discrimination ability [6, 31, 32]. Unexpectedly, behavioral monitoring during open field exploration revealed that *Prep1*^*i/+*^mice feature a significantly reduced locomotor activity (hypokinesia). This behavior might be related to constitutional expression of Prep1 in the cerebral cortex, which is reduced in our mice. Indeed, we have observed that Prep1 is expressed in Calbindin-ir interneurons in superficial layers of the cortex (II–III), which represent a class of slow-spiking interneurons. Interestingly, hypokinesia, cortical neurodegeneration, and alterations in odor preference behavior are all present in Alzheimer’s disease. Moreover, subcortical hypokinesia and alterations in odor preference behavior are also present in neurodegenerative phenotypes of Parkinson disease and Huntington disease [33, 34], suggesting that impairment of olfactory-based information processing might arise from degenerative mechanisms that mostly affect higher cortical regions and limbic area. Previous studies, indeed, described that the olfactory bulb is implicated in certain types of olfactory learning and memory, and that the natural replacement of bulbar interneurons may be programmed to occur after the transferal of the memories held by these neurons to other parts of the brain [35]. However, this is unlikely to occur in *Prep1*^*i/+*^ mice because the immunofluorescence for astrocytes (marked with S-100 protein) did not show any sign of astrogliosis, which usually accompany neurodegenerative phenomena. On the other hand, although we do not demonstrate a direct effect of Prep1 on the development of the olfactory bulb, it is largely recognized that two Prep1 co-factors, Pbx1 and Meis2, play key roles in olfactory bulb formation. More specifically, Pbx1 and Meis2, which belong to the same family of Prep1 (TALE proteins), are necessary for the neurogenesis of periglomerular cells [20, 21]. Therefore, the absence of astrogliosis and the role in neurogenesis of Prep1 co-factors strongly suggest that *Prep1*^*i/+*^ mice might display deficit in OB development rather than a neurodegeneration-induced dysfunction of this brain region.

Since olfactory perceptions define a series of neuroendocrine signals which regulate general metabolic homeostasis, the morphofunctional defects observed in olfactory system of *Prep1*^i/+^ mice suggest a profound impact on animal feeding behavior. Therefore, to evaluate alterations in olfactory-mediated eating behavior, mice underwent a food choice test for assessing their ability to choose between standard and high-fat food, which is generally more attractive to animals [36]. Intriguingly, *Prep1*^*i/+*^ mice featured no significant preference for high-fat food, probably due to their altered olfactory discrimination capabilities. Indeed, recent reports indicate that olfactory bulbectomized rodents display deficits in appetitively mediated behavior [37]. Therefore, it is possible to hypothesize that impaired olfactory perception featured by *Prep1*^i/+^ mice mimics a satiety status which, in turn, favors peripheral nutrient utilization, which is consistently observed in our previous studies [11–14]. The feeding behavior is also regulated by other brain regions, such as the hypothalamus and the mesencephalic motivational system (dopamine neurons). However, it is unlikely that the alteration in food preference observed in *Prep1*^*i*/+^ mice derives from a direct effect of this protein on the hypothalamus or dopamine system because immunofluorescence mapping of Prep1 shows no detectable levels in these regions.

Affection of olfactory ability and anatomic–cytological alterations in OB of *Prep1*^i/+^ mice led us to investigate the possibility that these phenotypes may be linked to an impairment of neurotrophin signaling on olfactory plasticity. Several authors have demonstrated that TrkB receptor exerts BDNF-mediated signaling for appropriate migration, differentiation, and proliferation of neuroblasts within the olfactory area, via ERK1/2 kinase, influencing the olfactory function [24, 25, 38]. *Prep1*^*i/+*^ mice olfactory bulbs display significantly reduced TrkB expression and activation of the main second messenger, ERK kinase. These observations seem to be in agreement with previous evidences in TrkB knock-down mice and TrkB adult-born neurons knockout mice, which display impaired spontaneous olfactory discrimination ability and decreased locomotor activity [39]. To further verify that the observed phenotypes are attributable to the different action/expression of Prep1, we have transfected neuronal cell line (N2A) with a *Prep1* cDNA, which mirrors the opposite situation occurring in mouse model. Consistently, N2A overexpressing Prep1 displayed a significant increase of TrkB mRNA and protein expression compared to control cells. In parallel, also the responsiveness to BDNF stimulation was found increased in N2A^*Prep1*^, with higher ERK kinase phosphorylation levels and increased cell viability.

## Conclusion

Although further investigations are needed for conclusion to be drawn, results of this study give a first knowledge on the role of the homeobox *Prep1* gene in brain tissue, strengthening the hypothesis that Prep1 is able to favor neuronal cell functionality by controlling BDNF-TrkB-mediated neurotrophic signaling pathway. To the best of our knowledge, no previous evidence has underlined association between Prep1 and adult neuronal cell responsiveness to BDNF-TrkB neurotrophic stimuli, as well as brain anatomical features and animal behavior. Thus, this study highlights a novel potential CNS marker associated to the impaired olfaction-mediated eating behavior observed in metabolic disorders.

## Electronic supplementary material

Supplemental Fig. 1 (GIF 502 kb)

High resolution image (TIFF 30931 kb)

Supplemental Fig. 2 (GIF 148 kb)

High resolution image (TIFF 9673 kb)

Supplemental Fig. 3 (GIF 149 kb)

High resolution image (TIFF 13826 kb)

## Abbreviations

BDNF: brain-derived neurotrophic factor
CB: cerebellum
COX: cytochrome *C* oxidase
CX: cortex
EPL: external plexiform layer
ERK: extracellular signal-regulated kinases
GCL: granule cell layer
GL: glomerular layer
GR: granular layer
HIPP: hippocampus
HYP: hypothalamus
IPL: internal plexiform layer
ir: immunoreactive
MCL: mitral cell layer
MOB: main olfactory bulb
OSNs: olfactory sensory neurons
OB: olfactory bulb
ONL: olfactory nerve layer
OPL: outer plexiform layer
PGC: periglomerular cells
Prep1: Pbx regulating protein 1
SVZ: subventricular-zone
RMS: rostral-migratory stream
TrkB: tropomyosin receptor kinase B
