# Supplementary figures and images for: Prep1 Deficiency Affects Olfactory Perception and Feeding Behavior by Impairing BDNF-TrkB Mediated Neurotrophic Signaling
Source: Mol Neurobiol. 2018 Jan 18;55(8):6801–15. doi: 10.1007/s12035-018-0873-7 (PMC6061220; doi:10.1007/s12035-018-0873-7)

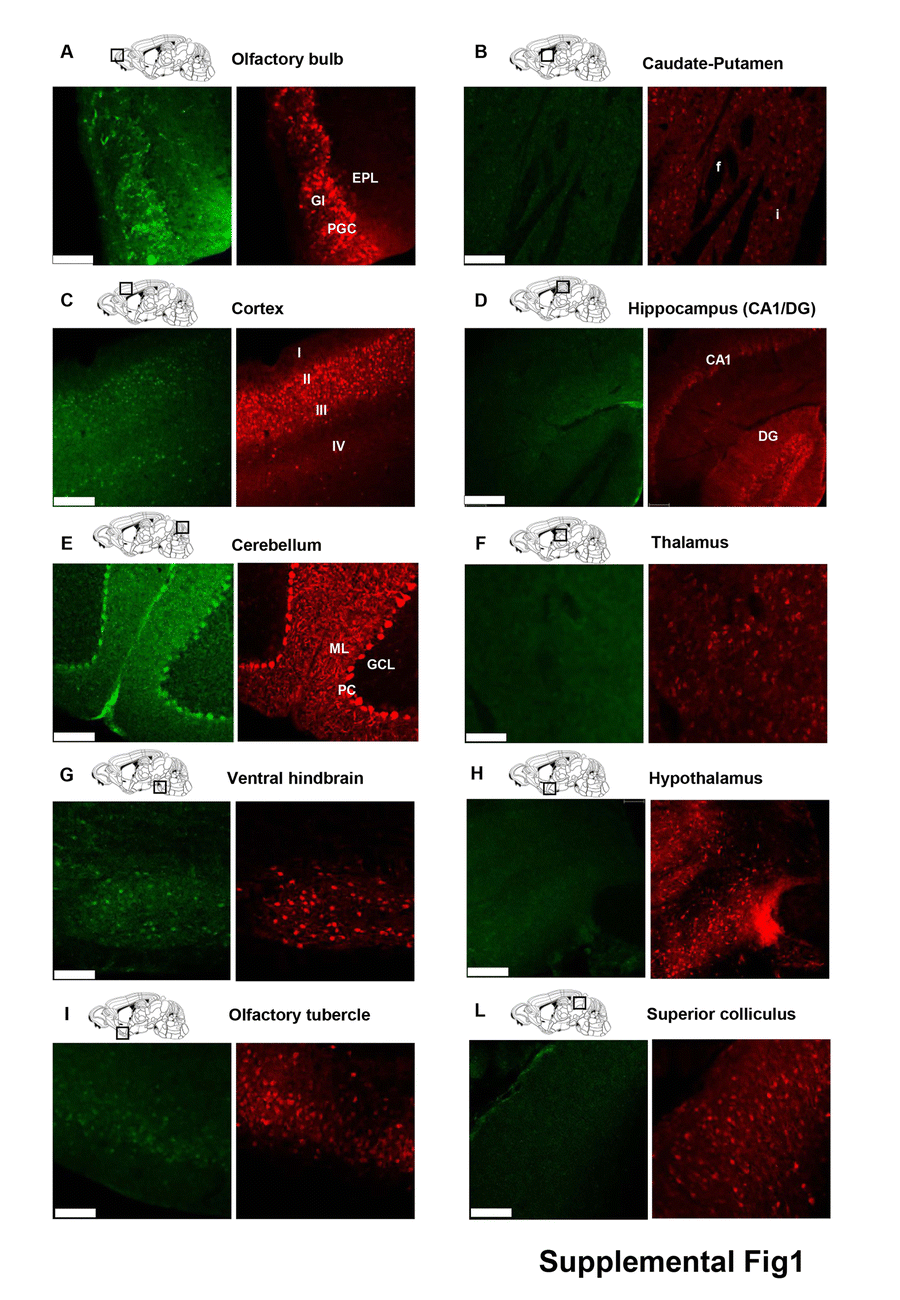

Supplement: Supplementary file 1 — (GIF 502 kb) [file 12035_2018_873_Fig8_ESM.gif]

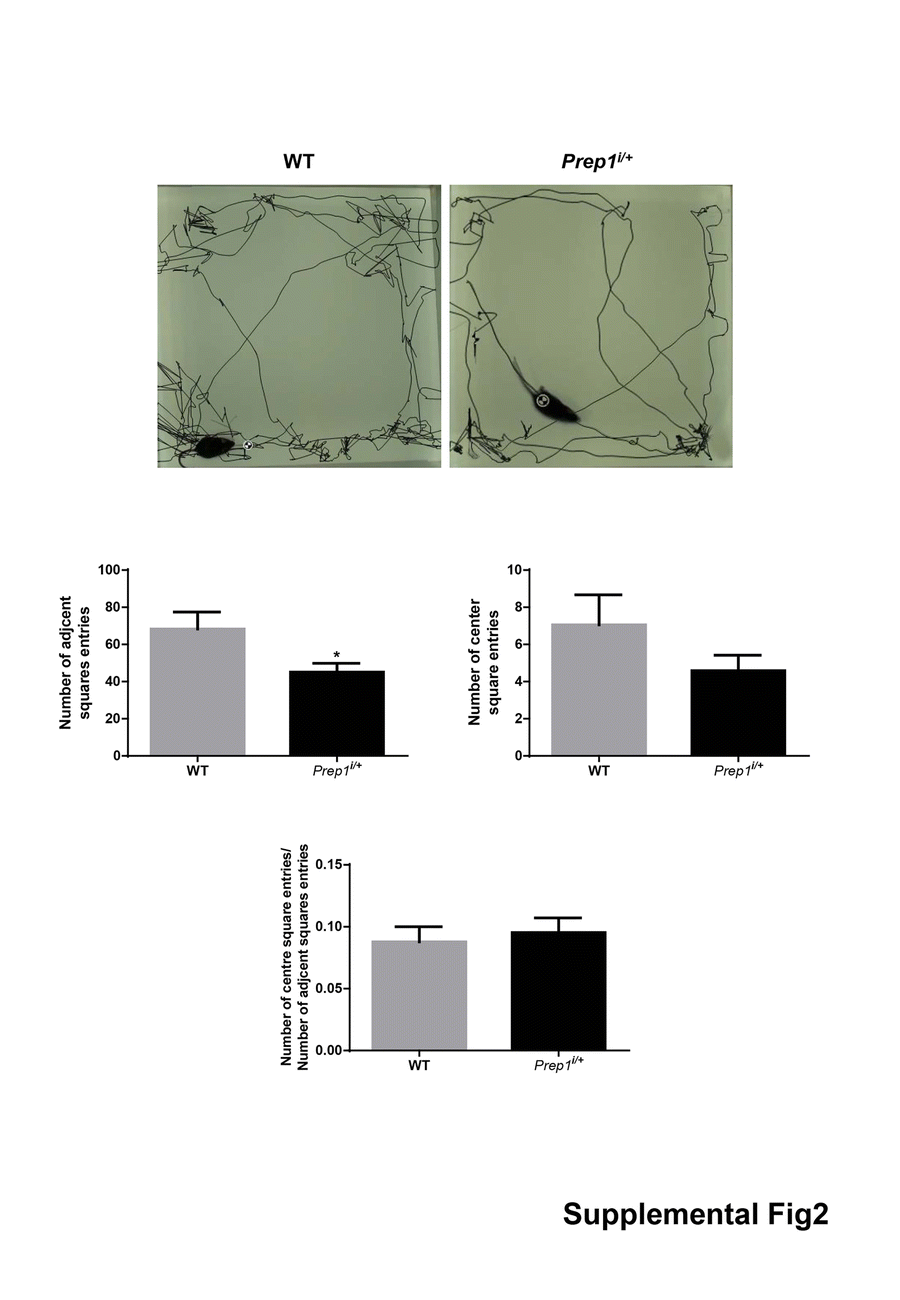

Supplement: Supplementary file 3 — (GIF 148 kb) [file 12035_2018_873_Fig9_ESM.gif]

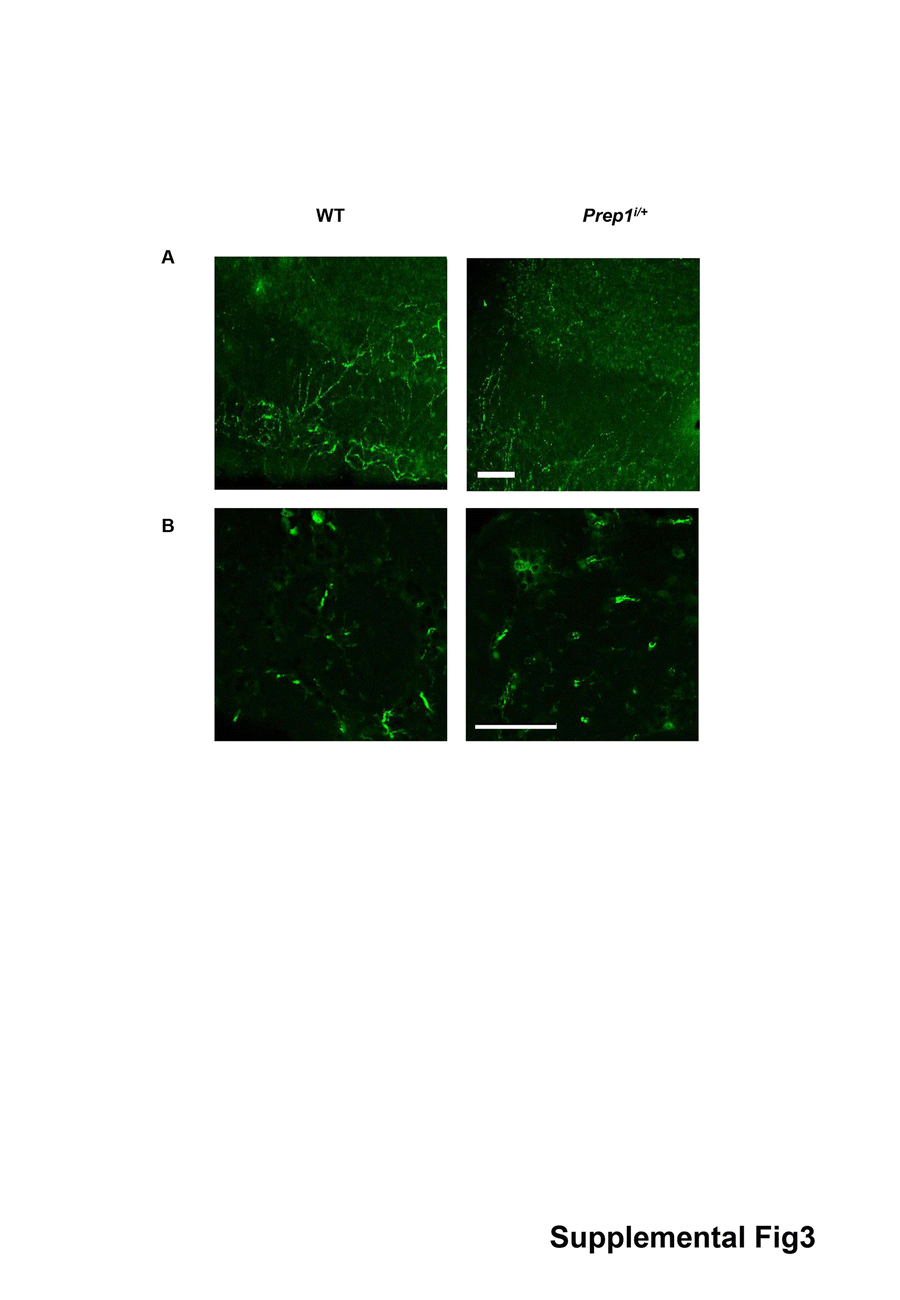

Supplement: Supplementary file 5 — (GIF 149 kb) [file 12035_2018_873_Fig10_ESM.gif]
